# Artificial cellulose derivatives are metabolized by select human gut Bacteroidota upon priming with common plant β-glucans

**DOI:** 10.1128/jb.00198-25

**Published:** 2025-07-21

**Authors:** Deepesh Panwar, William A. Stewart, Andrew Rodd, Harry Brumer

**Affiliations:** 1 Michael Smith Laboratories, University of British Columbia, Vancouver, British Columbia, Canada; 2 Department of Biochemistry and Molecular Biology, University of British Columbia 175086, Vancouver, British Columbia, Canada; 3 Department of Chemistry, University of British Columbia 428105, Vancouver, British Columbia, Canada; 4 Department of Botany, University of British Columbia 98685, Vancouver, British Columbia, Canada; National Institutes of Health, Bethesda, Maryland, USA

**Keywords:** human gut microbiota, *Segatella*, *Bacteroides*, cellulose ethers, cellulose derivatives, xyloglucan, mixed-linkage β-glucan, Bacteroidota, food, human diet

## Abstract

Synthetic ethers of cellulose (β(1,4)-glucan) are widely used in the food and pharmaceutical industry as thickeners, gelling agents, emulsifiers, and stabilizers. Consequently, humans ingest significant amounts of artificial cellulose derivatives in diets containing processed foods and through oral drug formulations. In the present study, we examined the potential of mixed-linkage β-glucan (MLG) and xyloglucan (XyG) polysaccharide utilization loci of autochthonous human gut (gastrointestinal tract) Bacteroidota to enable metabolism of artificial cellulose derivatives, based on the commonality of their backbone linkages. Two representative *Bacteroides* and six representative *Segatella* (syn. *Prevotella*) strains all failed to grow on carboxymethyl cellulose (CMC, E466), methyl cellulose (MC, E461), hydroxypropyl methyl cellulose (HPMC, E464), and hydroxyethyl cellulose (HEC) as sole carbohydrate sources. However, remarkably, collateral metabolism of cellulose ethers was observed when bacteria were primed with low levels of cereal MLG or dicot XyG, in a species-dependent, strain-dependent, and polysaccharide-dependent manner. Using the type strain *Segatella copri* DSM18205 as an example, cellulose derivative utilization was rationalized by demonstrating that outer membrane-localized *endo*-glucanases were both transcriptionally upregulated and possessed side activities toward CMC, MC, HPMC, and/or HEC. On one hand, our results *in vitro* counter the conventional wisdom that soluble cellulose derivatives are non-metabolizable in the human gut. On the other hand, our study suggests that broader analysis of this underappreciated metabolic ability is warranted in a wider range of taxa, especially in consideration of potential physiological effects in the context of balanced diets comprising plant polysaccharides.

**IMPORTANCE:**

Our data reveal a previously unknown potential among members of the human gut microbiota to metabolize artificial cellulose derivatives used in processed food and oral pharmaceuticals, which is driven by plant glycans ubiquitous in well-balanced diets containing natural dietary fiber. These results challenge the conventional wisdom that cellulose ethers are not broken down and metabolized in monogastric animals and motivate broader exploration of this phenomenon across the numerous autochthonous taxa.

## INTRODUCTION

**Fig 1:**
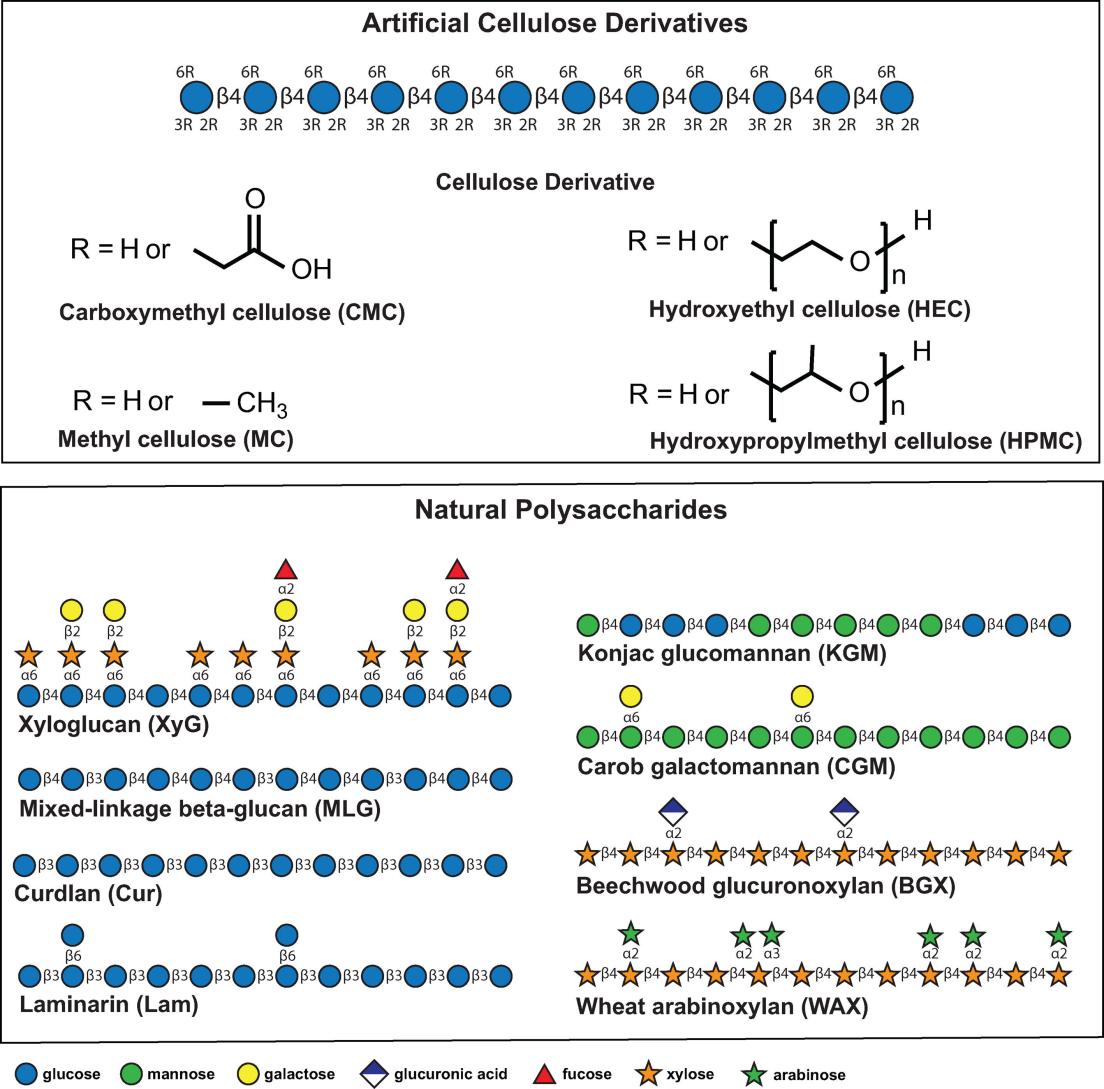
Artificial cellulose derivatives and natural plant polysaccharides. Monosaccharides and linkages are represented using the Symbol Nomenclature for Glycans (https://www.ncbi.nlm.nih.gov/glycans/snfg.html).

Chemically modified cellulose derivatives (Fig. 1) are water-soluble hydrocolloids that are widely used in the food and pharmaceutical industry as thickeners, gelling agents, emulsifiers, and stabilizers. Approved food additives (1) include methyl cellulose (MC; E461 in the International Numbering System for Food Additives), ethyl cellulose (E462), hydroxypropyl cellulose (HPC, E463), hydroxypropyl methyl cellulose (HPMC, E464), methyl ethyl cellulose (MEC, E465), carboxymethyl cellulose (CMC, “cellulose gum,” E466; also crosslinked E468), and ethyl hydroxyethyl cellulose (E467). These additives are found in diverse processed foods, including ketchup, salad dressing, gravy, fruit pie filling, dairy products, soft drinks, liquid meal replacements, meat substitutes, and baked goods (1–5). Films of modified celluloses, especially CMC, are of particular contemporary interest as “edible packaging” (6). CMC is also ubiquitous in commercial toothpastes widely used for dental hygiene. Diverse cellulose ethers are common components of oral drug formulations (7).

As indicated by their names, these artificial derivatives are ethers comprising different alkyl substituents and variable degrees of substitution of the hydroxyl groups on the β(1,4)-glucan polysaccharide chain (8). Despite the prevalence of artificial cellulose derivatives in food and oral pharmaceuticals, our understanding of the potential of members of the human gut microbiota (HGM) to metabolize these glucose-based polysaccharides is essentially unknown (9–14). Indeed, the conventional wisdom is that cellulose ethers are non-digestible and non-metabolizable (15–18).

Bacteroidota (syn. Bacteroidetes) comprise one of the two major phyla of the HGM post-weaning, together with Bacillota (syn. Firmicutes) (19). Previously, we functionally characterized specific polysaccharide utilization loci (PULs [20]) from human gut Bacteroidota, which confer individual *Bacteroides* and *Segatella* (syn. *Prevotella*) species with the ability to grow on the amorphous plant cell wall β-glucans, xyloglucan (XyG), and mixed-linkage β-glucan (MLG) (21–26). Notably, the vanguard outer membrane *endo*-glucanases of these systems often have demonstrable, albeit low, activity on CMC and HEC, due to structural commonality of the β(1,4)-glucan backbones between the natural and artificial polysaccharides.

Hence, we hypothesized that these side activities might be sufficient to enable the utilization of artificial cellulose derivatives by XyG-PUL-containing and MLG-PUL-containing Bacteroidota. We demonstrate, here, that none of the *Bacteroides* and *Segatella* strains tested were able to grow on artificial cellulose derivatives as sole carbohydrate sources *in vitro*. Remarkably, however, the addition of growth-limiting amounts of MLG or XyG “primed” several human gut *Segatella* species to metabolize artificial cellulose derivatives. Where observed, growth was concordant with specific *endo*-glucanase gene upregulation and biochemical activity. Our study thus reveals a metabolic potential for artificial cellulose derivatives that was previously unknown among Bacteroidota of the HGM, and which is driven by natural plant glycans. These results indicate that chemically modified cellulose-based hydrocolloids in food and pharmaceuticals are likely to be metabolized by human gut *Segatella*, in the context of a normal diet that includes plant-based dietary fiber (27).

## RESULTS

### Plant β-glucans prime human gut *Segatella* for cellulose derivative metabolism

*Bacteroides ovatus* ATCC 8483 (21, 22), *Bacteroides uniformis* ATCC 8492 (23, 28), and *Segatella copri* DSM18205 each contain experimentally validated MLG and XyG PULs (25, 26), which include biochemically defined outer membrane *endo*-glucanases or *endo*-xyloglucanases from glycoside hydrolase families GH16 and GH5 (see Fig. S1 for a graphical reference of all genes discussed in the text below). Furthermore, our previous metagenome analysis has revealed that these bacteria and their corresponding PULs are highly common in humans (21–23, 26).

**Fig 2:**
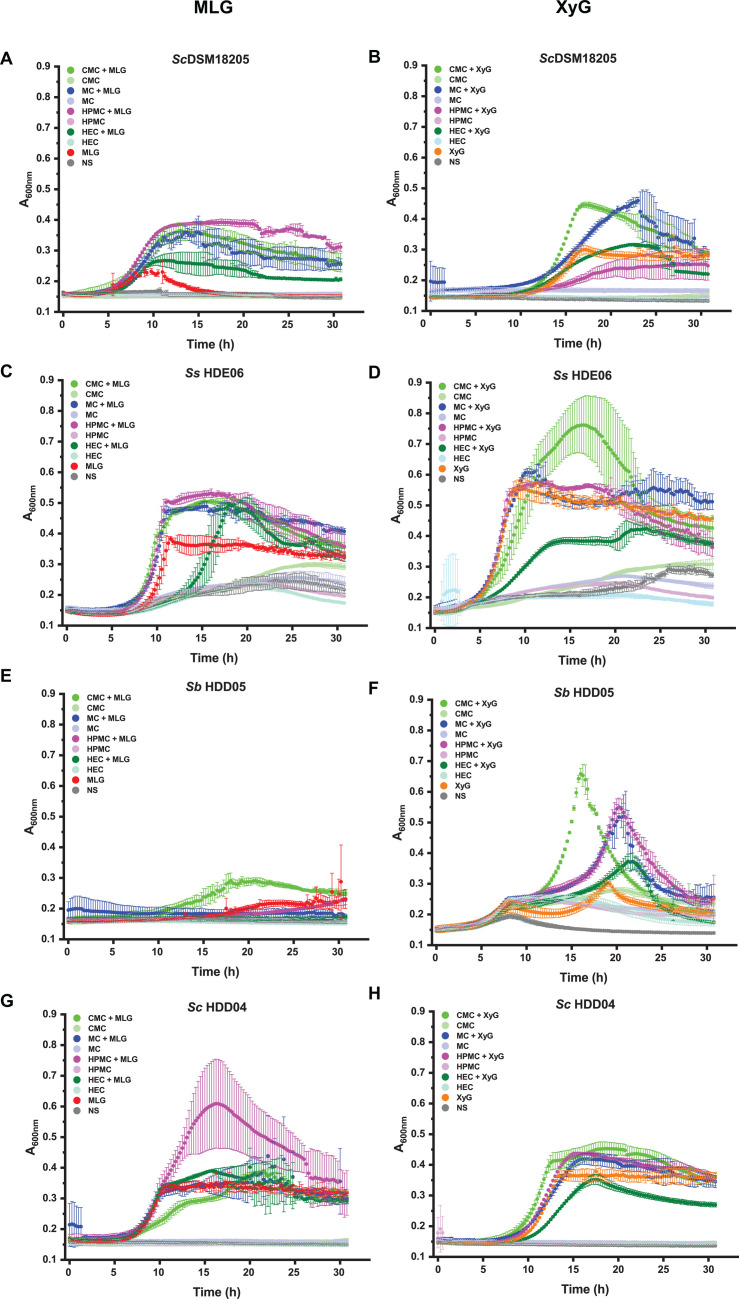
Growth of *Segatella* strains on MLG, XyG, and artificial cellulose derivatives. (**A–H**) Optical density of *S. copri* DSM18205, *S. sinica* HDE06, *S. brasiliensis* HDD05, and *S. copri* HDD04 liquid cultures containing combinations of MLG, XyG, CMC, HEC, MC, and HPMC, as indicated in individual panel titles and keys. The concentration of MLG or XyG was 0.5 mg/mL, and cellulose derivatives were 4.5 mg/mL each. Error bars indicate standard deviations of means for biological replicates (*n* = 3).

To explore the possibility that these β-glucan PULs might also enable the utilization of cellulose derivatives, we first screened these strains and several additional *Segatella* HGM isolates for growth on CMC, HEC, MC, and HPMC (see Fig. 1 for structures). *B. ovatus* ATCC 8483, *B. uniformis* ATCC 8492, *S. copri* DSM18205, *Segatella sinica* HDE06, *S. copri* HDA04, *S. copri* HDD04, *Segatella hominis* HDD12, and *Segatella brasiliensis* HDD05 all failed to grow directly on any of the artificial cellulose derivatives (4.5 g/L) as the sole carbohydrate source (Fig. 2; Fig. S2). For comparison, these strains generally grew well in 0.5 g/L MLG or XyG, despite being present at ninefold lower concentration.

We then tested whether this growth-limiting concentration of these natural polysaccharides (21–23, 25) could induce the collateral utilization of artificial cellulose derivatives. Strikingly, we observed that when *S. copri* DSM18205, *S. sinica* HDE06, *S. brasiliensis* HDD05, or *S. copri* HDD04 were incubated with 4.5 g/L of individual cellulose ethers in the presence of either 0.5 g/L MLG or XyG, a large growth potentiation was observed in many cases (Fig. 2).

For example, incubation of *S. copri* DSM18205 with MLG plus CMC, HPMC, MC, or HEC resulted in much higher *A*_600_ values at the end of the exponential growth phase than obtained with 0.5 g/L MLG alone (Fig. 2A). Likewise, XyG at 0.5 g/L resulted in greatly increased growth of *S. copri* DSM18205 on CMC and MC, whereas the effect on HEC was limited and HPMC appeared to be inhibitory (Fig. 2B). In the case of *S. sinica* HDE06, the presence of MLG (Fig. 2C) enabled synergistic growth on CMC, HPMC, MC, and HEC (although with a notable increase in the lag phase with MLG plus HEC). With XyG (Fig. 2D), enhanced growth of *S. sinica* HDE06 was only observed for CMC, but to a more limited extent. Growth on HPMC or MC plus XyG was essentially unchanged, and HEC again appeared to be inhibitory, versus growth on the natural polysaccharide alone. A synergistic growth effect was also observed with *S. brasiliensis* HDD05 for CMC plus MLG (Fig. 2E), and for CMC, MC, HPMC, or HEC plus XyG (Fig. 2F). Likewise, the growth of *S. copri* HDD04 was potentiated slightly on the combinations HPMC plus MLG (Fig. 2G), CMC plus XyG, MC plus XyG, and HPMC plus XyG, whereas HEC inhibited growth on XyG (Fig. 2H).

Furthermore, we also observed that growth potentiation was titratable, using *S. copri* DSM18205 and *S. sinica* HDE06 as examples. Total bacterial growth on MLG or XyG as the sole carbohydrate source was dependent on polysaccharide concentration down to the lowest value tested, 0.0625 g/L (Fig. S3 and S4, compare with Fig. 2). In the case of *S. copri* DSM18205, growth was barely detectable at this concentration with both MLG and XyG (maximum Δ*A*_600_ ca. 0.1). Yet in all cases, for both *S. copri* DSM18205 and *S. sinica* HDE06, significant concentration-dependent growth potentiation was observed when MLG or XyG were added to 4.5 g/L CMC, MC, and HPMC. Likewise, MLG potentiated the growth of *S. copri* DSM18205 on HEC. On the other hand, HEC appeared to inhibit the growth of *S. sinica* HDE06, dependent on the concentration of MLG or XyG. Taken together, these data demonstrate that the natural plant β-glucans MLG and XyG prime human gut *Segatella* to break down and metabolize synthetic cellulose ethers, in a species-dependent, strain-dependent, and derivative-dependent manner.

### Specific *endo*-β-(xylo)glucanases are implicated in cellulose derivative breakdown

The growth analyses suggest that the metabolism of artificial cellulose derivatives is dependent upon induction of the MLG-PULs and XyG-PULs by their corresponding substrates. In the absence of this induction, i.e., on cellulose derivatives alone, no growth occurs. Rapid upregulation of individual PULs in response to substrate sensing is well known (29, 30), and we have specifically demonstrated this for the MLG-PULs and XyG-PULs of *S. copri* DSM18205 (25). Hence, we used *S. copri* DSM18205 to explore further the substrate promiscuity of the corresponding *endo*-β-(xylo)glucanases to hydrolyze the β-glucan backbone of cellulose ethers.

**Fig 3:**
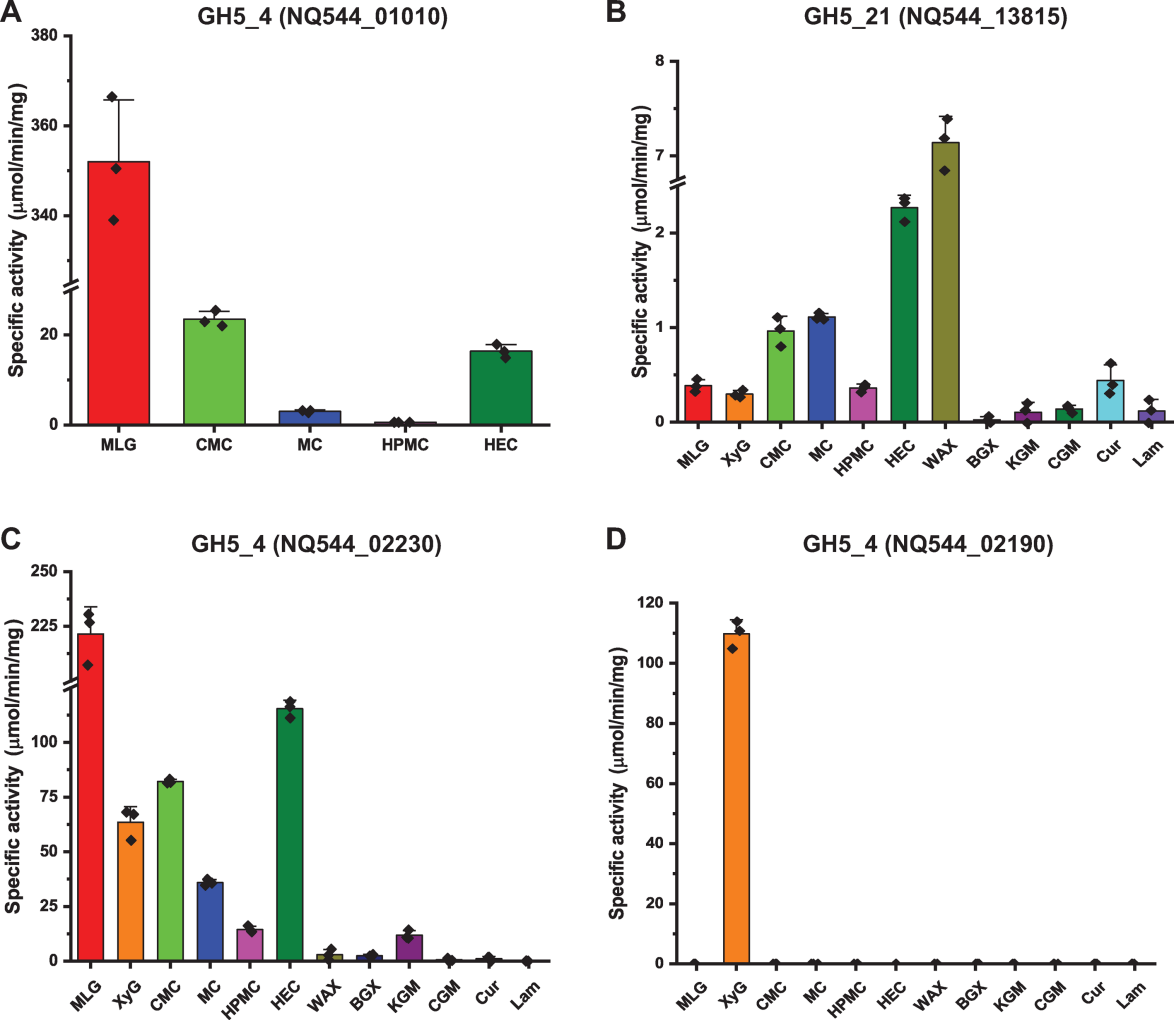
Specific activities of *S. copri* DSM 18205 endo-glycanases toward natural and artificial polysaccharides. (**A**) GH5_4 member encoded by locus NQ544_01010 in the MLG-PUL. This analysis extends our previous characterization of this enzyme (26) to include CMC, MC, HPMC, and HEC; the assay on MLG was repeated as a reference. (**B**) GH5_21 member encoded by locus NQ544_13815. (**C**) GH5_4 member encoded by locus NQ544_02230. (**D**) GH5_4 member encoded by locus NQ544_02190 in the XyG-PUL. The enzymes in panels B to D have not been previously characterized and were therefore subjected to a broad panel of plant cell wall polysaccharides to determine their overall substrate ranges. See Fig. S1 for a graphical overview of gene loci. Error bars represent standard deviation of means for biological replicates (*n* = 3).

We extended our previous biochemical characterization (26) of the vanguard outer membrane GH5_4 MLGase from the *S. copri* DSM18205 MLG-PUL (encoded by gene locus NQ544_01010, Fig. S1) by testing activity on CMC, MC, HPMC, and HEC. Although low compared to MLG, weak specific activity was detected on CMC, MC, and HEC (Fig. 3A). This activity was concordant with the hypothesis that upregulation of the MLG-PUL by growth-limiting amounts of MLG enabled collateral utilization of these derivatives (Fig. 2). Surprisingly, specific activity was extremely low on HPMC, despite this derivative exhibiting the highest growth potentiation (Fig. 3A). This prompted us to perform a targeted transcriptional analysis to identify other potentially co-regulated GH5 homologs that might provide HPMC-cleaving activity.

**Fig 4:**
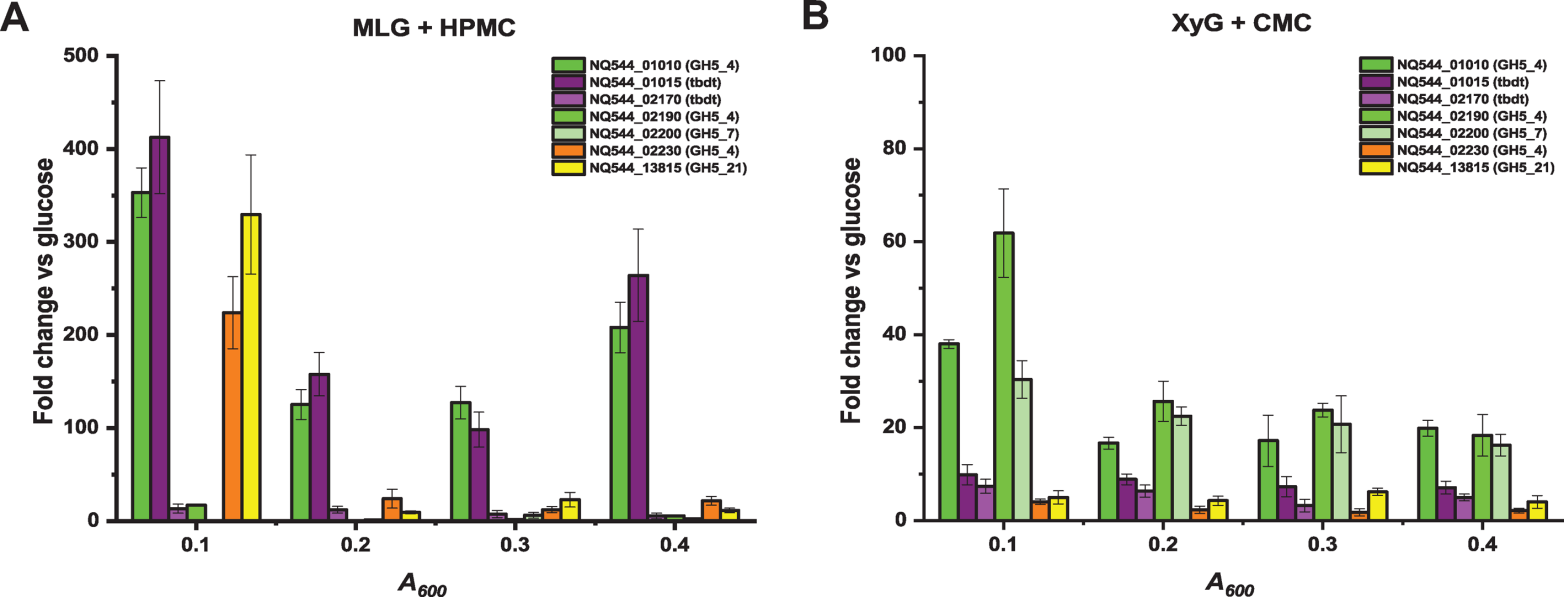
RT-qPCR analysis of GH5 members and related genes in *S. copri* DSM18205. *S. copri* DSM18205 RNA was isolated at optical densities (*A*_600_) ranging from 0.1 to 0.4 which corresponds to early-exponential to mid-exponential growth. Genes encoding TonB-dependent transporters (*tbdt*) of the MLG-PUL and XyG-PUL, as well as GH5_4 and GH5_7 members adjacent to the XyG-PUL, were also included for reference. (**A**) *S. copri* DSM18205 grown in the presence of MLG plus HPMC at different *A*_600_ was compared with cells grown in the presence of glucose until mid-log phase. (**B**) *S. copri* DSM18205 grown in the presence of XyG plus CMC at different *A*_600nm_ was compared with cells grown in the presence of glucose until mid-log phase. Error bars represent standard error of means of two biological and two technical replicates. See Fig. S1 for a graphical overview of gene loci.

Reverse transcription-quantitative polymerase chain reaction (RT-qPCR) analysis (Fig. 4A) during the growth of *S. copri* DSM18205 on MLG plus HPMC revealed high upregulation of genes encoding the GH5_4 member (locus NQ544_01010) and the TBDT (locus NQ544_01015) from the MLG-PUL during exponential growth versus glucose-grown control, as expected (25). For comparison, genes encoding the outer membrane GH5_4 member (locus NQ544_02190) and associated TBDT (locus NQ544_02170) from the XyG-PUL were only weakly transcribed and not differentially regulated over the growth phase, as expected (25). Notably, however, genes encoding an independent GH5_4 member (locus NQ544_02230) and a xylan-PUL-associated (31) GH5_21 member (locus NQ544_13815) were transiently upregulated very early in the exponential phase (Fig. 4A).

Subsequent biochemical analysis of the recombinant GH5_21 member revealed predominant activity on wheat arabinoxylan over a range of natural glycans, weak activity on CMC, MC, and HEC, and very limited activity on HPMC (Fig. 3B). On the other hand, the GH5_4 member encoded by locus NQ544_02230 had the highest activity on MLG and strong activity on XyG, CMC, MC, and HEC, with specific activity values ca. twofold to sixfold less than that on MLG (Fig. 3C). This activity profile is similar to an ortholog from the rumen isolate *Segatella bryantii* (*Prevotella bryantii*) B_1_4 (57% amino acid sequence identity, 71% similarity), for which activity on MLG (primary), XyG, CMC, and HEC was demonstrated (32). Although comparatively low among the range of polysaccharides tested, the specific activity of the NQ544_02230 GH5_4 member on HPMC (ca. 13 µmol/minute/mg) was notably higher than that observed for the MLG-PUL GH5_4 (locus NQ544_01010, Fig. 3A) and the xylan-PUL GH5_21 (locus NQ544_13815, Fig. 3B). Full Michaelis-Menten kinetic analysis of the NQ544_02230 GH5_4 member recapitulated the substrate profile observed by specific activity measurements and showed that MLG, CMC, MC, and HMPC all had similar *K*_m_ values while the artificial cellulose derivatives had much lower *k*_cat_ values; HEC showed uniquely high *K*_m_ and *k*_cat_ values (Fig. S5 and Table S1). These data suggest that the GH5_4 MLGase/XyGase encoded by locus NQ544_02230 may provide sufficient activity to enable the growth of *S. copri* DSM18205 on HPMC following induction by MLG.

Regarding the utilization of artificial cellulose derivatives following inclusion of XyG in the medium, we examined the activity profile of the recombinant outer membrane GH5_4 member encoded by locus NQ544_02190 in the XyG-PUL of *S. copri* DSM18205 (25). As expected, the predominant activity of this enzyme was toward xyloglucan (Fig. 3D). In comparison, specific activity toward HEC, MC, CMC, and HPMC (in descending order) was extremely weak and only quantifiable by extending the assay time to 9.5 hours using 100-fold higher enzyme concentration than used for XyG. This again prompted us to use transcript analysis to reveal potential complementary enzymes.

RT-qPCR of *S. copri* DSM18205 during growth on XyG plus CMC (Fig. 4B) showed that locus NQ544_02190 (Fig. S1D), which encodes the core GH5_4 *endo*-xyloglucanase of the XyG-PUL, was upregulated on this mixture, as expected based on our previous RNAseq analysis using pure XyG (25). Notably, locus NQ544_01010 (Fig. S1C), which encodes the predominant mixed-linkage *endo*-glucanase of the MLG-PUL (26), was also upregulated early in exponential-phase growth on the XyG/CMC mixture, albeit to a lesser degree (Fig. 4B). The demonstrable activity of the MLG-PUL GH5_4 member on CMC, as well as MC, provides a parsimonious explanation for the growth potentiation of *S. copri* DSM18205 on mixtures of these soluble cellulose derivatives with XyG (Fig. S1B). Interestingly, we did not observe upregulation on XyG plus CMC of NQ544_02230 (Fig. 4B), which encodes the multi-functional GH5_4 (Fig. 3C), that explained the growth potentiation observed with cellulose ethers and MLG (see above). We also observed that locus NQ544_02200, which encodes a GH5_7 member, was upregulated to a similar extent as locus NQ544_01010 of the MLG-PUL (Fig. 4B). GH5_7 members have been exclusively shown to act on β-*manno*-configured substrates (mannan and mannosides, see https://www.cazy.org/GH5_7_characterized.html [33, 34]), so activity on cellulose derivatives is highly unlikely. Together with our previous study (25), these gene expression data suggest that there is a general derepression of diverse CAZymes in the stationary and early exponential phases of growth. Thus, it is possible that other, yet unidentified, *endo*-glycanases in *S. copri* DSM 18205 also contribute to the utilization of artificial cellulose derivatives in the presence of natural polysaccharides.

## DISCUSSION

Despite the prevalence of artificial cellulose derivatives in food, there appears to be little information available on the potential of these polysaccharides to be utilized by the HGM for growth. This contrasts with our understanding of the metabolism of insoluble crystalline cellulose fibers from plant cell walls, which has been studied at the levels of consortia, individual species, and specific molecular systems of bacteria in humans and other animals (10, 13, 35–39). Indeed, our understanding of the microbiological degradation of cellulose ethers appears to be limited to early studies on soil bacteria and fungi (40).

Here, we show that several key HGM members from the phylum Bacteroidota are unable to grow on soluble cellulose ethers as a sole carbohydrate source. Yet remarkably, some strains in the genus *Segatella* (family *Prevotellaceae*, phylum Bacteroidota) are clearly able to utilize individual cellulose derivatives, but only when incubated together with the natural plant cell wall matrix β-glucans MLG and XyG. Our previous (25, 26) and current transcriptional analysis, together with biochemical data, directly implicate the production of individual *endo*-β-glucanases/*endo*-β-xyloglucanases in this ability.

In this context, it is interesting to note that studies on the archetypal PUL system, the starch utilization system (Sus), have revealed that outer membrane-localized *endo*-glycanases are present in very low levels at the cell surface of Bacteroidota, even in the absence of the cognate polysaccharide (30, 41). These “surveillance” levels of enzyme are essential because PUL upregulation is mediated by individual sensor/regulator proteins in the periplasm, which require that short polysaccharide breakdown products (oligosaccharides) transit the outer membrane through PUL-encoded TonB-dependent transporters (SusC homologs) for sensing (29, 30). From our data, we may conclude that either the initial level of enzyme activity of MLG-PUL and XyG-PUL *endo*-(xylo)glucanases toward cellulose derivatives is too low, and/or transport and sensing of fragment oligosaccharides are insufficient, to enable growth on pure cellulose ethers. However, the introduction of even low amounts of the natural PUL substrates triggers production and delivery of additional *endo*-glycanase molecules to the cell surface. When this occurs, the side activities of these enzymes toward artificial cellulose derivatives lead to their breakdown, import, and subsequent catabolism.

Our data also show that strain-to-strain differences exist, despite the presence of syntenic PULs and enzymes (Fig. S1). Illuminating the molecular bases for these differences will require further detailed study of the specificities of glycosidases, transporters, and downstream metabolic enzymes. In this context, two things are worth noting. First, as introduced above, soluble cellulose ethers are heterogeneous with regard to their regiochemistry and degree of substitution, as a consequence of their industrial syntheses (4, 8, 40). Thus, it is difficult to ascertain exactly how much glucose might be accessed directly, or after potential cleavage of ether substituents, by the bacterial cell. Maximum cell densities in 4.5 g/L of individual cellulose derivatives (Fig. 2; Fig. S3) did not reach levels obtained with similar concentrations of natural β-glucans like MLG or XyG (21–23, 25), in which all of the monosaccharides are presumably accessible. This suggests that only a fraction of the glucose in artificial cellulose derivatives is available for metabolism. Yet, we note that MLG and XyG also cause upregulation of genes encoding a GH3 β-glucosidase and a GH94 cellobiose phosphorylase in *S. copri* DSM 18205 (25, 26), which are likely candidates enabling further saccharification of imported cellulose-ether fragments. Second, cellulose ethers appear to be inhibitory in some cases, resulting in extended lag phases and/or reduced total cell density versus growth on MLG or XyG alone. The molecular mechanism(s) of this inhibition will likewise require further study. Regardless, our data indicate that artificial cellulose derivatives exert direct metabolic effects on common members of the HGM in combination with normal dietary polysaccharides. With increasing interest in the nexus of food additives, the HGM, and disease, we highlight that the potential for taxon-specific metabolism of artificial cellulose derivatives should be considered in addition to their physicochemical effects as polymers (9–14).

We focused here on the unexpected potentiation of soluble artificial cellulose derivative utilization by taxa in the phylum Bacteroidota that are not classically associated with native, insoluble cellulose breakdown. Foremost in HGM, *Ruminococcus* species (phylum Bacillota, syn. Firmicutes) are noteworthy for their ability to utilize crystalline cellulose (35, 39). Furthermore, it can be reasonably expected that the diverse range of cellulolytic enzymes in these bacteria (35) may also degrade soluble artificial cellulose derivatives in the diet. Indeed, CMC is a widely used proxy substrate for recombinant cellulases, including those from ruminococci (35, 42, 43). However, to the best of our knowledge, it remains to be studied whether these cellulolytic Bacillota can grow on soluble cellulose derivatives directly, or whether they may also require native plant cellulose or other cell wall glycans for induction. Likewise, direct utilization of synthetic cellulose ethers by “cellulolytic” *Bacteroides* (44) and other taxa (35) from the HGM remains to be studied.

Thus, we emphasize that our present study only provides a first glimpse into cellulose derivative utilization in the HGM, yet motivates broader exploration of this phenomenon across the numerous autochthonous taxa. At the same time, our data challenge the conventional wisdom that cellulose ethers, e.g., CMC and HPMC, are non-metabolizable in monogastric animals such as humans (15–18). Certainly, these polymers are poorly broken down, hence their well-known laxative properties when taken in large amounts (45). Dietary exposure data are sparse, but it has been reported that adults in the United States ingest an average of 25 mg/kg/day of CMC (E466) alone (1, 15), which may be used in food up to 2% wt/wt (12). Plant β-glucans, including MLG and XyG, are common in human diets (24), suggesting that the priming mechanism we observed may also be relevant in the HGM, leading to metabolism of diverse artificial cellulose ethers routinely ingested in processed foods. Further combined nutritional and microbiological studies will be required, of course, to determine the physiological relevance of cellulose ether metabolism in people. We note that the content of plant β-glucans in the diet, the presence of individual taxa, and their specific enzymology will be important co-variables to consider in both humans and model systems (9–14, 16–18, 46, 47).

## MATERIALS AND METHODS

### Carbohydrates

Barley mixed-linkage β-glucan (P-BGBL), tamarind xyloglucan (P-XYGLN), wheat arabinoxylan (P-WAXYL), beechwood xylan (P-XYLNBE), konjac glucomannan (P-GLCML), carob galactomannan (P-GALMH), and *Alcaligenes faecalis* curdlan (P-CURDL) were obtained from Megazyme. *Eisenia bicyclis* laminarin (YL02421) was procured from Biosynth. Glucose (BDH9230) was sourced from VWR, carboxymethyl cellulose (AC332641000) from Fisher Scientific, hydroxyethyl cellulose (K391) from Amresco, and both methyl cellulose (M0512) and (hydroxypropyl)methyl cellulose (H9262) from Sigma.

### Bacterial strains and growth media

*B. ovatus* ATCC 8483 and *B. uniformis* ATCC 8492 were generously provided by Professor Eric Martens (University of Michigan, Ann Arbor). *Segatella copri* DSM18205, *S. copri* HDD04, *S. copri* HDA04, *S. hominis* HDD12, *S. sinica* HDE06, and *S. brasiliensis* HDD05 (48, 49) were obtained from the Leibniz Institute DSMZ—German Collection of Microorganisms. All strains were cultured at 37°C in an anaerobic chamber (Coy Laboratory Products Inc., Grass Lake, MI, USA) with an atmosphere consisting of 90% N_₂_, 5% CO_₂_, and 5% H_₂_. Modified peptone yeast glucose (mPYG) medium was used to propagate all *Segatella* and *Bacteroides* strains, except *S. sinica* HDE06 for which modified yeast casitone fatty acid (mYCFA) medium was used, as described previously (25). All media were filter sterilized and allowed to equilibrate in the chamber for at least 6 hours before use.

### Bacterial growth measurements

Bacterial strains were propagated in freshly prepared mPYG or mYCFA media, as indicated above, overnight. Cells were pelleted by centrifugation, washed with 2× mYCFA, and resuspended to achieve a final optical density (*A*_600_) of 0.08. To test growth on cellulose derivatives, 90 µL of sterilized 10 g/L CMC, MC, HPMC, or HEC was mixed with 10 µL of sterile water. Alternatively, 10 µL of either MLG or XyG, prepared in a twofold serial dilution ranging from 10 to 1.25 g/L, was added in place of water. Subsequently, 100 µL of 2× mYCFA containing the bacterial suspensions was added to the mixture in a flat-bottom 96-well plate, resulting in a final volume of 200 µL. This corresponds to initial conditions of 4.5 g/L of cellulose derivatives, a concentration gradient of MLG or XyG ranging from 0.5 to 0.0625 g/L, and a cell density (*A*_600_ value) of 0.04. Plates were sealed and placed in a microplate reader (PowerWave HT, Biotek Instruments, Winooski, VT, USA), where the *A*_600_ was recorded every 15 minutes over 30 hours, with 10 seconds of shaking prior to each reading. All growth experiments were performed in biological triplicates.

### Transcriptional profiling by RT-qPCR

RNA isolation and RT-qPCR were performed essentially as described previously (25). *S*. copri DSM18205 was propagated in mPYM, followed by washing and resuspension in 2× mYCFA devoid of carbohydrate. Thereafter, 2× mYCFA containing *S. copri* DSM18205 at *A*_600_ ~ 0.08 was added to a 2× solution of MLG plus HPMC or XyG plus CMC in a 1:1 ratio, resulting in a final volume of 20 mL (final concentrations: MLG or XyG, 1 g/L and HPMC or CMC, 2 g/L; cells, *A*_600_ ~ 0.04). One milliliter sample from each biological replicate was harvested at *A*_600_ values ranging from 0.1 to 0.4 and stored at −70°C for RNA isolation. The primers for individual genes (Table S2) were designed using the Primer3 software. Primer efficiency values (Table S2) were calculated from the slope of plots of Cq values versus log(gDNA concentration), using the equation: *E* = −1 + 10^(−1/slope)^ × 100%. Transcript levels at each time point were calculated using the *ΔΔCt* method and normalized to transcript levels in *S. copri* DSM18205 grown in glucose until mid-log phase; *recA* was used as a housekeeping gene control.

### Recombinant enzyme production

The MLG PUL GH5_4 (NQ544_01010) was produced and purified by immobilized metal-ion affinity chromatography as described previously (26) (Fig. S6). GH5_4 (NQ544_02230), GH5_21 (NQ544_13815), and GH5_4 (NQ544_02190) genes minus predicted signal peptides were cloned in vector pMCSG53 through ligation-independent cloning and transformed into *Escherichia coli* BL21(DE3). Cells were grown to *A*_600_ of 0.6 in lysogeny broth containing 100 µg/mL ampicillin and induced with 0.5 mM Isopropyl β-ᴅ-1-thiogalactopyranoside (IPTG), followed by overnight incubation at 16°C and 200 rpm. Cells were harvested via centrifugation at 6,000 × *g* for 30 minutes at 4°C and were subjected to sonication in lysis buffer: 20 mM phosphate buffer, pH 7.4, 300 mM NaCl (150 mM NaCl for NQ544_02190). Lysed cells were centrifuged at 10,000 × *g* for 15 minutes at 4°C, and the supernatant was affinity-purified on a Bio-Rad NGC Chromatography System. A 5 mL HisTrap HP column (Cytiva) was equilibrated with lysis buffer, and the supernatant was loaded onto the column, followed by washing with 25 mL of lysis buffer. His-tagged proteins were eluted by applying a linear gradient of elution buffer: 20 mM sodium phosphate, pH 7.4, 400 mM imidazole, 300 mM NaCl (150 mM NaCl for NQ544_02190) over 50 mL. SDS-PAGE was used to identify pure fractions for pooling, which were combined and concentrated (Fig. S6). GH5_4 (NQ544_02230) and GH5_21 (NQ544_13815) were stored in 20 mM sodium phosphate buffer, pH 6.0, 300 mM NaCl, while NQ544_02190 was stored in 50 mM citrate buffer, pH 6.0, 50 mM NaCl. Protein concentrations were determined from *A*_280_ measurements on a BioTek Take3 (Agilent) microvolume plate reader, using calculated molar extinction coefficients from the ProtParam tool on the ExPASy server (50).

### Enzyme assays

Enzyme activities were determined using the copper-bicinchoninic acid (BCA) assay for reducing sugars (51). Assays were incubated at 37°C for 6 minutes for GH5_4 (NQ544_02230) and GH5_4 (NQ544_01010) or 10 minutes for GH5_21 (NQ544_13815) and GH5_4 (NQ544_02190). The incubation time of GH5_4 (NQ544_02190) was additionally extended to 9.5 hours to probe for trace activity on all substrates other than XyG. The reactions were stopped by the addition of 100 µL of BCA working reagent, and color was developed with a 20 minute incubation at 80°C.

The pH activity profiles of GH5_4 (NQ544_02230) and GH5_21 (NQ544_13815) (Fig. S7) were determined in glycine HCl (pH 3.0), citrate phosphate buffer (pH 4–5.5), phosphate buffer (pH 6.0–7.5), and glycine NaOH (pH 9.0) using the following procedure. Briefly, 10 µL of GH5_4 (NQ544_02230) at 1 µg/mL and 10 µL of MLG at 5 mg/mL in ultrapure water were mixed with 80 µL of respective buffers at 50 mM. Similarly, 10 µL of GH5_21 (NQ544_13815) at 10 µg/mL and 10 µL of WAX at 5 mg/mL in ultrapure water were mixed with 80 µL of respective buffers at 50 mM (Fig. S6A and B).

The pH activity profile of GH5_4 (NQ544_02190) (Fig. S7) was determined using citrate buffer (pH 3–6), phosphate buffer (pH 6–8), and glycine NaOH buffer (pH 9, 10). To start the reaction, 10 µL of enzyme in 0.1 mg/mL bovine serum albumin (BSA) was added to 90 µL of buffer/polysaccharide using a multichannel pipette. This 100 µL reaction contained 50 ng/mL GH5_4 (NQ544_02190), 0.01 mg/mL BSA, 50 mM buffer, and 2 mg/mL XyG (Fig. S6C).

The substrate specificities of GH5_4 (NQ544_02190) and GH5_4 (NQ544_01010) were determined using the same method as for the GH5_4 (NQ544_02190) pH activity profile. The 100 µL reaction contained 50 mM sodium citrate buffer at pH 6.0, 1.0 mg/mL polysaccharide, 0.01 mg/mL BSA, as well as between 10 ng/mL and 50 µg/mL GH5_4 (NQ544_02190) or GH5_4 (NQ544_01010). Enzyme concentration was optimized so that the number of reducing ends would fall within the range of the glucose standard curve. For GH5_4 (NQ544_02230) and GH5_21 (NQ544_13815), 10 µL of enzyme at different concentrations (1–50 µg/mL) was incubated in 50 mM phosphate buffer at pH 6.0 and 10 µL of substrates (5 or 10 g/L) as described in the pH activity profile.

Kinetic parameters of GH5_4 (NQ544_02230) on MLG or cellulose ethers were obtained using 10 µL of enzyme (0.5 or 1 µg/mL) added to 90 µL of substrate (0.1–7 g/L) in 45 mM of phosphate buffer at pH 6.0 and incubated for 6 minutes at 37°C. The Michaelis–Menten equation was fitted to initial-rate kinetic data using OriginPro Version 2025 (OriginLab Corporation, Northampton, MA, USA).

## Data Availability

All data associated with this article are presented in the main text and figures or supporting information.

## SUPPLEMENTAL MATERIAL

Supplemental tables and figures jb.00198-25-s0001.pdf Tables S1 and S2, and Fig. S1 to S7.

ASM does not own the copyrights to Supplemental Material that may be linked to, or accessed through, an article. The authors have granted ASM a non-exclusive, world-wide license to publish the Supplemental Material files. Please contact the corresponding author directly for reuse.
