# Supplementary material for: Artificial cellulose derivatives are metabolized by select human gut Bacteroidota upon priming with common plant β-glucans
Source: J Bacteriol. 2025 Jul 21;207(8):e00198-25. doi: 10.1128/jb.00198-25 (PMC12369377; doi:10.1128/jb.00198-25)
Supplement: Supplemental tables and figures — Tables S1 and S2, and Fig. S1 to S7. [file jb.00198-25-s0001.pdf]

Supplemental Tables

Table S1. ***S. copri*** **DSM18205 GH5_4 (NQ544_02230) Michaelis -Menten kinetics.***

**Substrate**	k_{cat} (s^{-1})	K_m (mg mL^{-1})	k_{cat}/K_m (s^{-1} mg^{-1} mL)
MLG	291 ± 25	0.35 ± 0.05	831
CMC	17± 4.9	0.26 ± 0.20	65
MC	16 ± 1.4	0.16 ± 0.04	100
HPMC	9.1 ± 1.1	0.41 ± 0.10	22
HEC	160± 19	0.60 ± 0.10	270

* Obtained by fitting the classic Michaelis-Menten equation to the data in Figure S5. $[E]_t$ for MLG, CMC, and HEC was 0.001185 µM and for MC and HPMC was 0.00237 µM.

Table S2. *S. copri* DSM 18205 Cloning and RT-qPCR primers.

Primer Name	Sequence (5'→3')	Use	Efficiency (%)
GH5_4_01010_F	CTTTCGCCGGTACCAATGAG	NQ544_01010 expression	99.3
GH5_4_01010_R	GTAAGATGAGGTGGCGCTTG	"	
MLG_TBDT_F	CGCTTGCTTACCCTCTATGC	NQ544_01015 expression	88
MLG_TBDT_R	ATGCCTTTACGGTTGACAGG	"	
XyG_ TBDT_F	AGAGAAGGGACATGCTGACC	NQ544_02170 expression	87
XyG_ TBDT_R	CAAAGTCCAACCGGCAGAAA	"	
GH5_4_02190_F	CGGAGTGCAGACCATCAATG	NQ544_02190 expression	95.4
GH5_4_02190_R	CTTCTTGGCACGGGTAATCG	"	
GH5_7_02200_F	TGCAGGTAAAGGACGGACAT	NQ544_02200 expression	94.8
GH5_7_02200_R	ACCTTGGTCTTCACTCCTCG	"	
GH5_4_02230_F	CAGATTGCCACCCGTTTCAA	NQ544_02230 expression	99.5
GH5_4_02230_R	GTCGTCACCATTAGCTGCAG	"	
GH5_21_13815_F	AAAGTCAGGTGCCGATGGTA	NQ544_13815 expression	90.5
GH5_21_13815_R	GCGGATGGGTAAGGGTCATA	"	
recA_F	GGTTCGCGTAAAGGTGGTAA	*recA* expression	103
recA_R	TATACCAGCTGCCGCTCTTT	"	
GH5_4_02230_F	TACTTCCAATCCAATGCAGAGAAAGTGCTGTGAATGCAGTGAAGAAC	NQ544_02230 catalytic domain cloning	
GH5_4_02230_R	TTATCCACTTCCAATGTTATTATTCATTATAAGCCTTTTTCATGGCTTCCACTACTGTAG	"	
GH5_21_13815_F	TACTTCCAATCCAATGCAGCCGATAATCTGCCGGCACTC	NQ544_13815 catalytic domain cloning	
GH5_21_13815_R	TTATCCACTTCCAATGTTATTATGTTGGATTTTGTTTCAAATACCACTCTTTATACC	"	
GH5_4_02190_F	TACTTCCAATCCAATGCAAGCTTCAAGGTTCTCAACCCAGAGC	NQ544_02190 full protein cloning	
GH5_4_02190_R	TTATCCACTTCCAATGTTATTATTTAGCAGGCCACTTTGCTGCTG	"	

Supplemental Figures

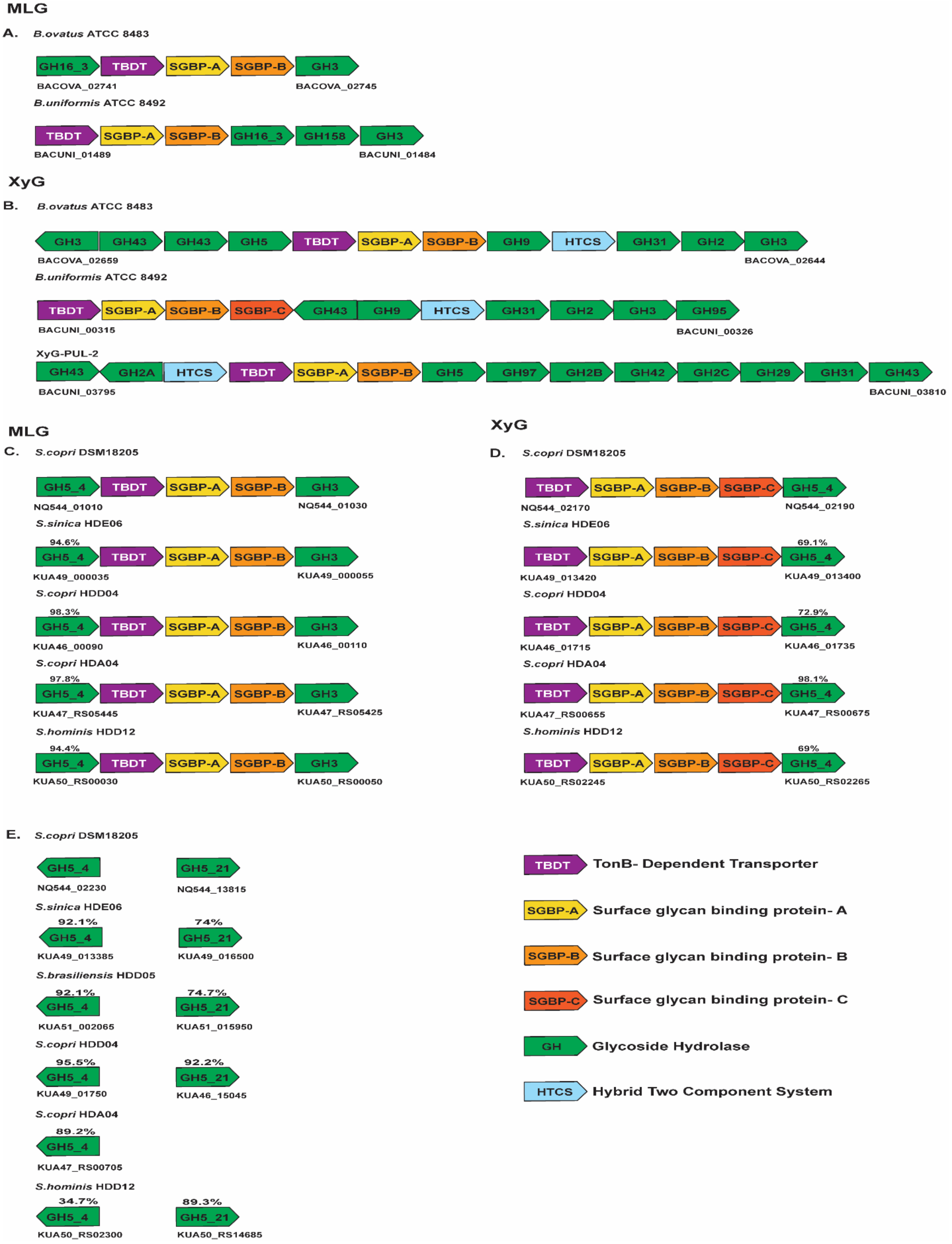

Figure S1. Homology of mixed-linkage β-glucan (MLG) and xyloglucan (XyG) Polysaccharide Utilization Loci (PULs), and other β-glycanase genes, among HGM bacteria in this study. (A) *Bacteroides* MLG-PULs, **(B)** *Bacteroides* XyG-PULs, **(C)** *Segatella* MLG-PULs, **(D)** *Segatella* XyG-PULs, **(E)** Other β-glycanases from *Segatella* species.

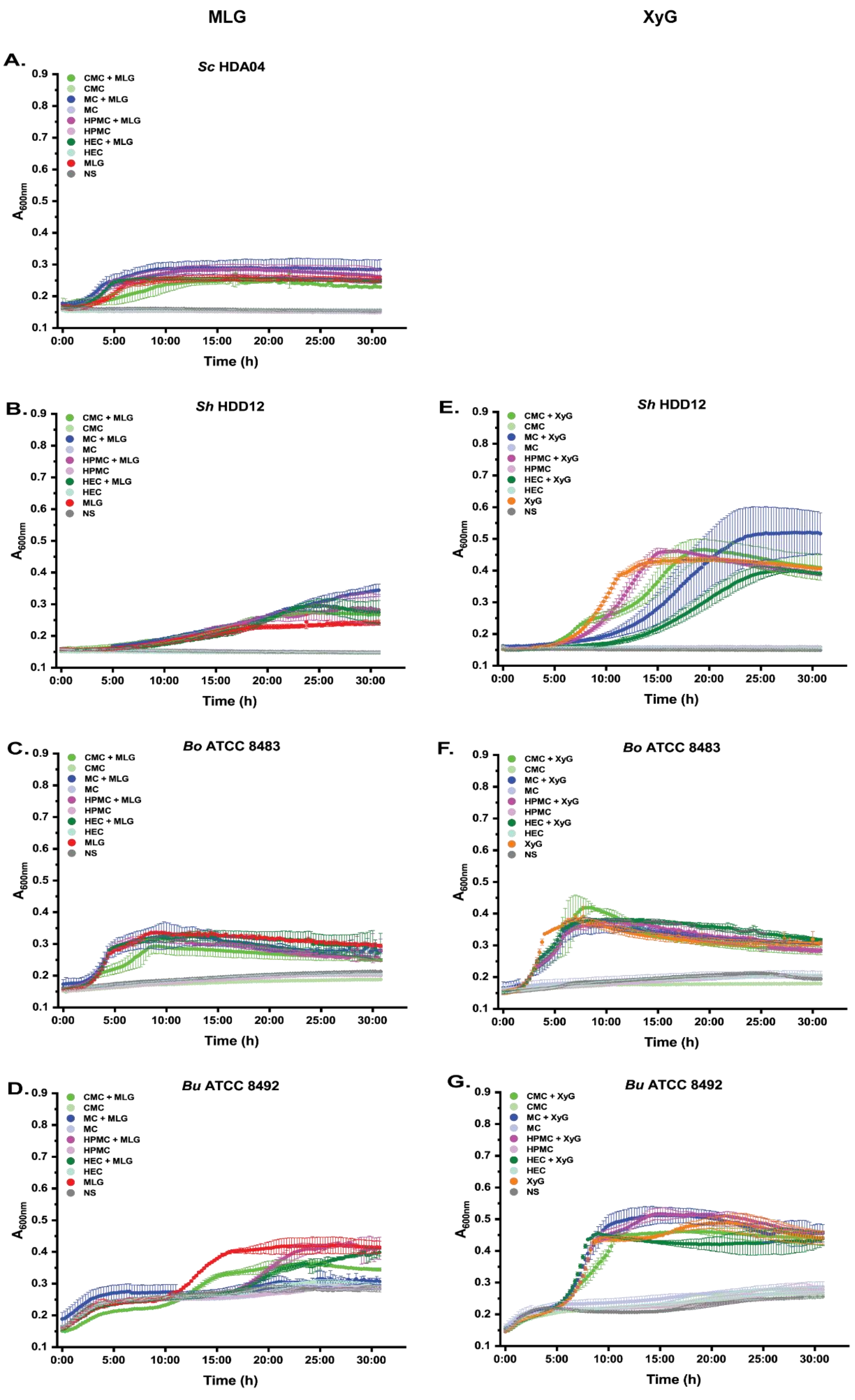
MLG
XyG
A.
Sc HDA04
B.
Sh HDD12
C.
Bo ATCC 8483
D.
Bu ATCC 8492
E.
Sh HDD12
F.
Bo ATCC 8483
G.
Bu ATCC 8492
CMC + MLG
CMC
MC + MLG
MC
HPMC + MLG
HPMC
HEC + MLG
HEC
MLG
NS
CMC + XyG
MC + XyG
HPMC + XyG
HEC + XyG
XyG
A_{600nm}
Time (h)
0.1
0.2
0.3
0.4
0.5
0.6
0.7
0.8
0.9
0:00
5:00
10:00
15:00
20:00
25:00
30:00

Figure S2. Growth of *Segatella* strains on MLG, XyG, and artificial cellulose derivatives. (**A**) *S. copri* HDA04 growth on cellulose derivatives plus MLG at 4.5 and 0.5 mg/mL respectively. (**B and E**) *S. hominis* HDD12 growth on cellulose derivatives plus MLG or XyG at 4.5 and 0.5 mg/mL respectively. (**C and F**) *B. ovatus* ATCC 8483 growth on cellulose derivatives plus MLG or XyG at 4.5 and 0.5 mg/mL respectively. (**D and G**), *B. uniformis* ATCC 8492 growth on cellulose derivatives plus MLG or XyG at 4.5 and 0.5 mg/mL respectively. Error bars indicate standard deviations of means for biological replicates (n = 3).

S. copri DSM18205

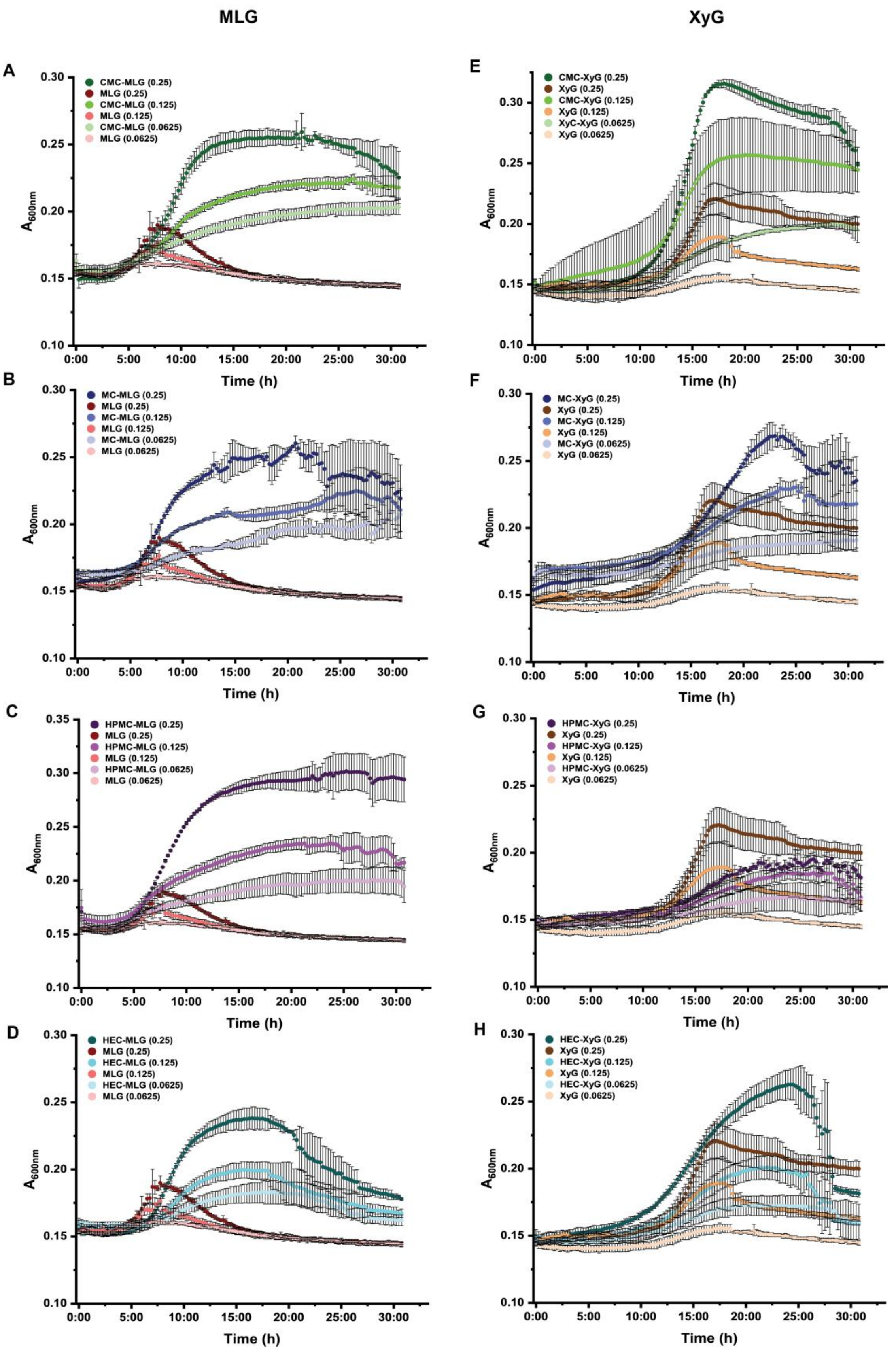

Figure S3. Growth of *S. copri* DSM18205 on artificial cellulose derivatives as a function of MLG or XyG concentration. (A–D): Growth curves of *S. copri* DSM 18205 showing the effect of MLG titration (0.25-0.0625 mg/mL) in the presence of individual cellulose derivatives (4.5 mg/mL). (E–H): Growth curves of *S. copri* DSM 18205 showing the effect of XyG titration (0.25-0.0625 mg/mL) in the presence of individual cellulose derivatives (4.5 mg/mL). The curves are colour coded as follows: CMC + MLG/XyG- green gradient; MC + MLG/XyG- blue gradient; HPMC + MLG/XyG- magenta gradient; HEC + MLG/XyG- cyan gradient. The controls are MLG alone- red gradient and XyG- orange gradient (0.25-0.0625 mg/mL). Error bars indicate standard deviations of means for biological replicates (n = 3).

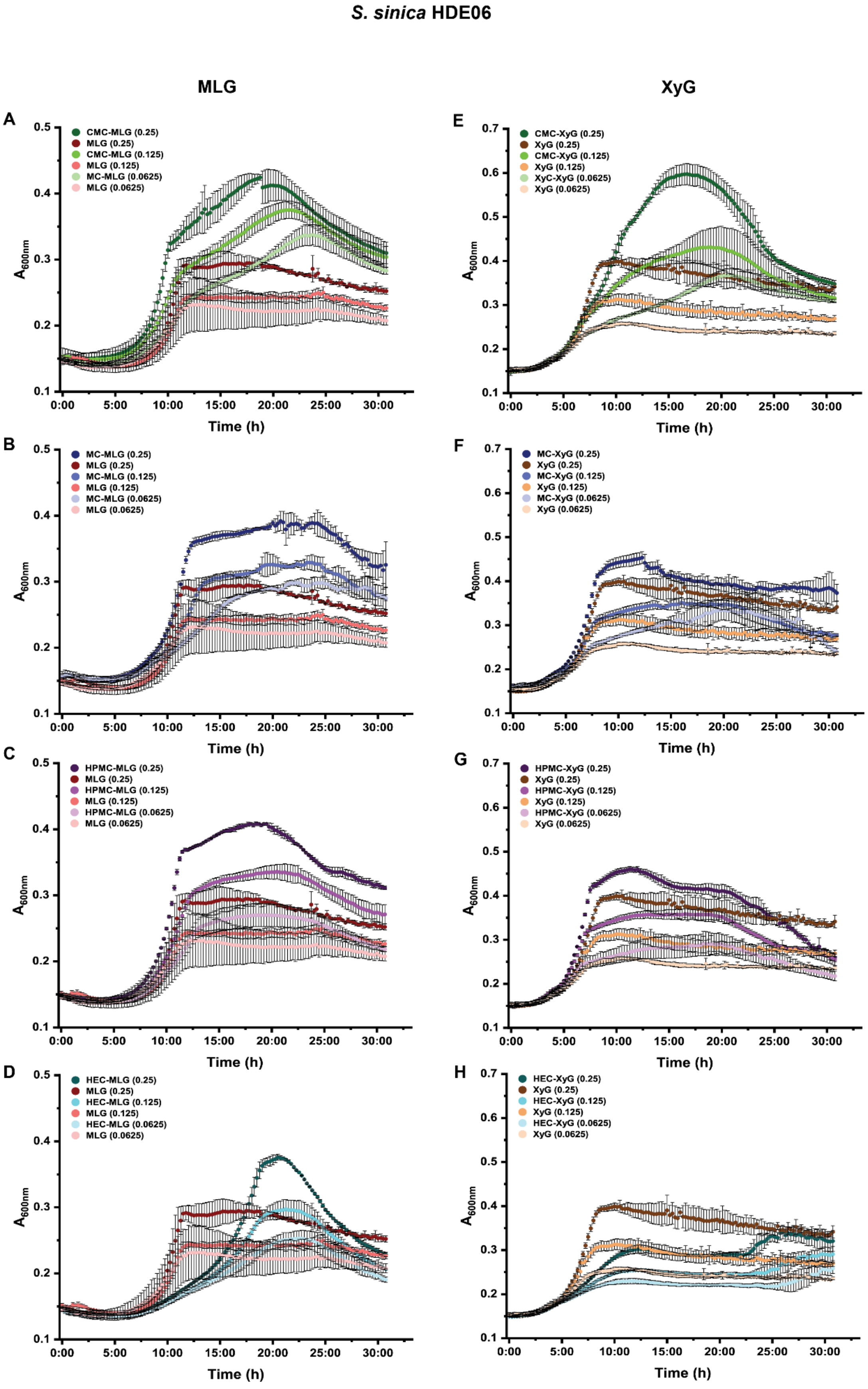
S. sinica HDE06
MLG
XyG
A
CMC-MLG (0.25)
MLG (0.25)
CMC-MLG (0.125)
MLG (0.125)
MC-MLG (0.0625)
MLG (0.0625)
E
CMC-XyG (0.25)
XyG (0.25)
CMC-XyG (0.125)
XyG (0.125)
XyC-XyG (0.0625)
XyG (0.0625)
B
MC-MLG (0.25)
MLG (0.25)
MC-MLG (0.125)
MLG (0.125)
MC-MLG (0.0625)
MLG (0.0625)
F
MC-XyG (0.25)
XyG (0.25)
MC-XyG (0.125)
XyG (0.125)
MC-XyG (0.0625)
XyG (0.0625)
C
HPMC-MLG (0.25)
MLG (0.25)
HPMC-MLG (0.125)
MLG (0.125)
HPMC-MLG (0.0625)
MLG (0.0625)
G
HPMC-XyG (0.25)
XyG (0.25)
HPMC-XyG (0.125)
XyG (0.125)
HPMC-XyG (0.0625)
XyG (0.0625)
D
HEC-MLG (0.25)
MLG (0.25)
HEC-MLG (0.125)
MLG (0.125)
HEC-MLG (0.0625)
MLG (0.0625)
H
HEC-XyG (0.25)
XyG (0.25)
HEC-XyG (0.125)
XyG (0.125)
HEC-XyG (0.0625)
XyG (0.0625)
A_{600nm}
Time (h)
0:00
5:00
10:00
15:00
20:00
25:00
30:00

Figure S4. Growth of *S. sinica* HDE06 on artificial cellulose derivatives as a function of MLG or XyG concentration. (**A**–**D**): Growth curves of *S. sinica* HDE06 showing the effect of MLG titration (0.25-0.0625 mg/mL) in the presence of individual cellulose derivatives (4.5 mg/mL). (**E**–**H**): Growth curves of *S. sinica* HDE06 showing the effect of XyG titration (0.25-0.0625 mg/mL) in the presence of individual cellulose derivatives (4.5 mg/mL). The curves are colour coded as follows: CMC + MLG/XyG- green gradient; MC + MLG/XyG- blue gradient; HPMC + MLG/XyG- magenta gradient; HEC + MLG/XyG- cyan gradient. The controls are MLG alone- red gradient and XyG- orange gradient (0.25-0.0625 mg/mL). Error bars indicate standard deviations of means for biological replicates (n = 3).

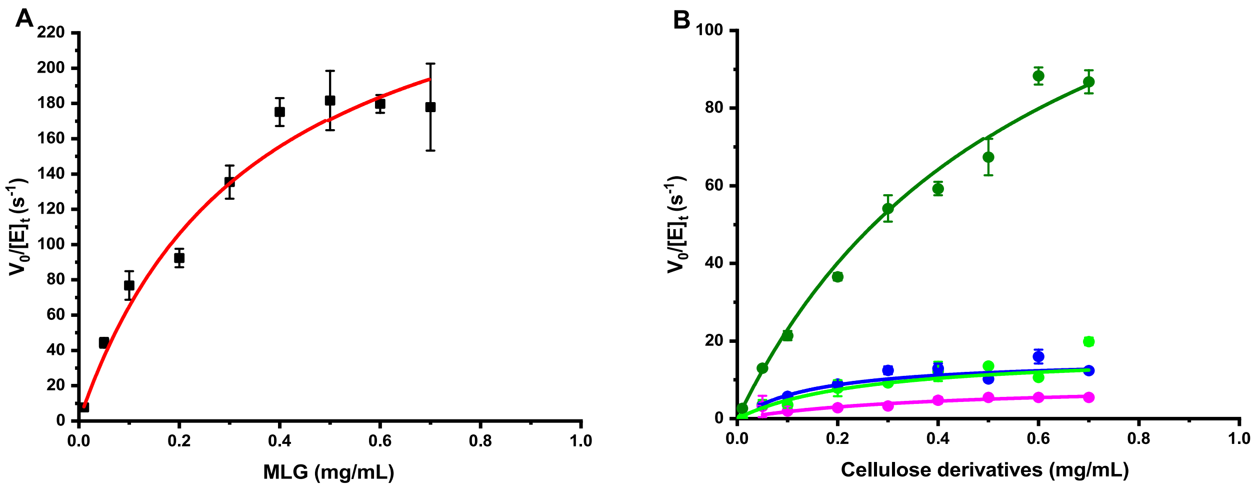

Figure S5. Michaelis-Menten kinetic analysis of the GH5_4 member encoded by locus NQ544_02230 of *S. copri* DSM18205. Kinetic parameters obtained by fitting the classic Michaelis-Menten equation to the data (coloured lines) are provided in Table S1. (**A**) MLG and (**B**) Cellulose derivatives (HEC, olive; CMC, green; MC, blue; and HPMC, magenta) Data were collected at the kinetic pH optimum, pH 6.0 (Figure S8). Error bars indicate standard deviations of means for biological replicates (n = 3).

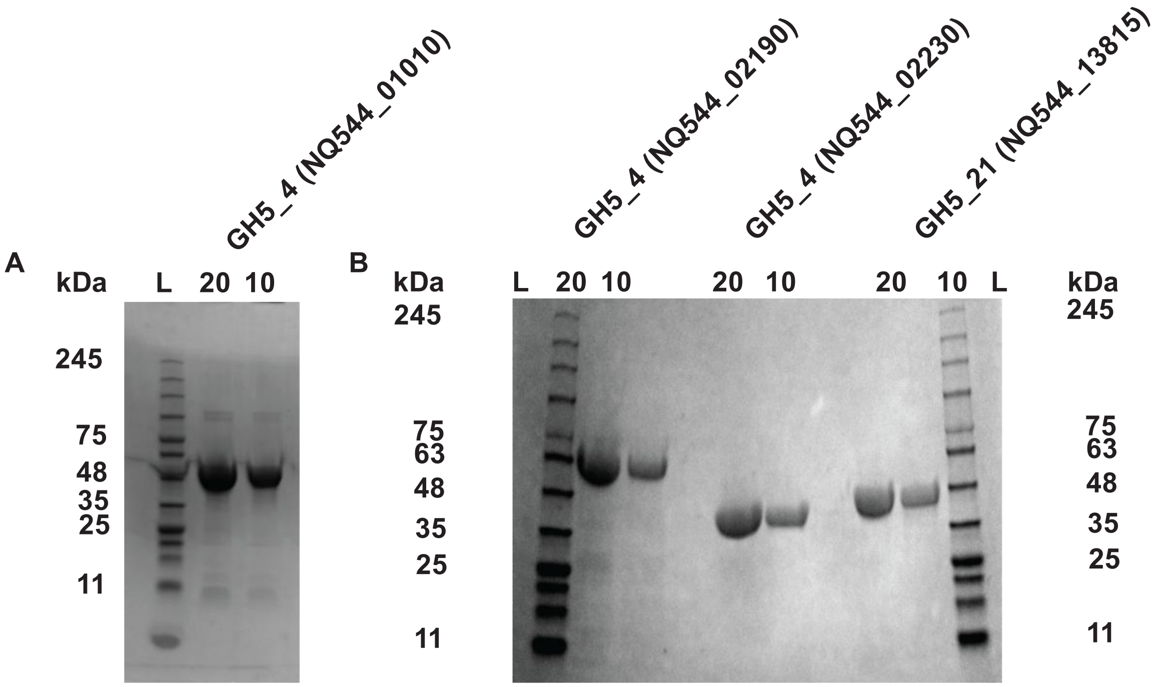

Figure S6. SDS-PAGE of expressed protein purified by metal affinity chromatography. (A) GH 5_4 (NQ544_01010). Calculated molecular weight, 43.8 kDa. (B) Other GH5 members. Calculated molecular weights: GH 5_4 (NQ544_02190), 55.7 kDa; GH 5_4 (NQ544_02230), 42.2 kDa; GH 5_21 (NQ544_13815), 47.3 kDa. As indicated at the top of the gels, proteins were loaded in two different amounts, 10 and 20 µg.

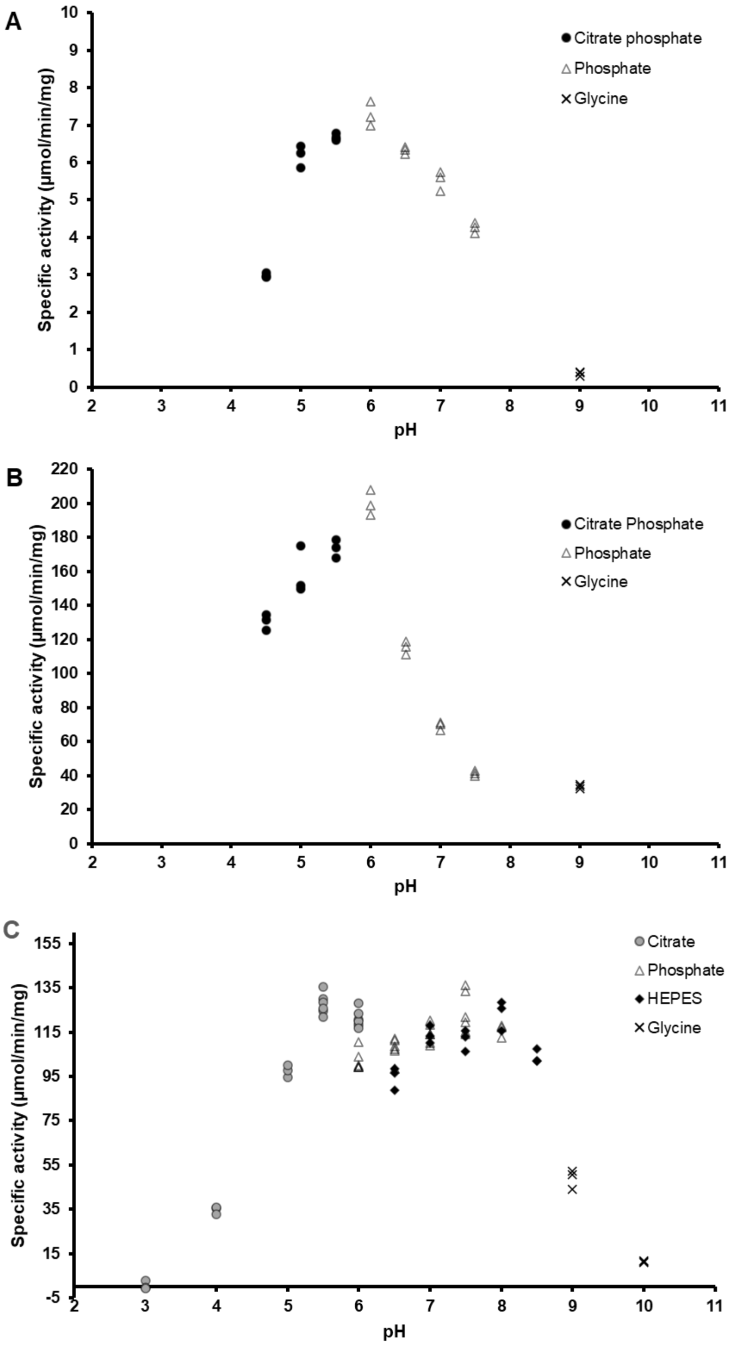

Figure S7. pH-activity profiles of of *S. copri* DSM18205 GH5 members. (**A**) GH5_21 member encoded by locus NQ544_13815. (**B**) GH5_4 encoded by locus NQ544_02230. (**C**) GH5_4 encoded by locus NQ544_02190 in the XyG-PUL. Standard curves containing 0-125 µM glucose in 50 mM buffer (citrate phosphate buffer pH 5.0, sodium citrate pH 6.0, sodium phosphate pH 6.0, HEPES pH 7.0, and glycine pH 9.0) where used to calculate the number of reducing ends in each condition. Error bars indicate standard deviations of means for biological replicates (n = 3).
